# 

**Published:** 1921-04-09

**Authors:** 


April 9, 1921] [Price Twopence

### The Hospital

The Workers' Newspaper of Administrative Medicine and Institutional Life, Administration, National Insurance and Health.

CONTENTS

The Cause of Medicine ... ... ... ... ... 23

A Public Warning ... ... ... ... ... 23

Dublin Castle and the Royal College of Surgeons 24

The Repair of Graves and Head-Stones ... ... 24

Notes of the Week ... ... ... ... ... 25
Hospitals and Approved Societies Funds—The Federation's Progress—Milk for Mothers—The Unemployed Miner—A Secretary on Paid Organisers—Two Large Charitable Distributions—The St. Mary's Units—Consultants for St. Pancras Poor-Law Hospital—St. Thomas's Hospital—"The Mundy Hospital"—Dentists for the Navy—What Contribution Schemes Can Do.

The Practice of Birth-Control ... ... ... 27

A Reflection.

Surgery in Otitis Cases ... ... ... ... ... 28
Some Valuable Notes for Practitioners.

Resident Operating Surgeons? ... ... ... 29

The Refuse Disposal of London ... ... ... 30

Annual Meetings of Hospitals ... ... ... ... 30

The Public Well-Being ... ... ... ... ... 31

Dr. Addison Goes—Caring for the Adolescent—The Doctor and the Strike.

The Editor's Letter-Box .. ... .. ... ... 33

"The Truth about Venereal Disease"—Should Nurses Smoke?

Passing Events and Topics ... ... ... ... 33

Nurses Want a Career ... ... ... ... 34

The Matrons' and Sisters' Department ... ... 35
The Pay of the Nurse.—V. A Comparison with Other Occupations.

Round the Hospitals ... ... ... ... 35

A New Light on Florence Nightingale ... ... 38

Matrons' and Sisters' Appointments ... ... 38

The College of Nursing (Limited by Guarantee) vi
What the Local Centres are Doing.

